# Self-protective behaviours to protect against coronavirus-19 infection and their association with Perceived Infectability, Germ Aversion and Fear of COVID-19 among South African learners

**DOI:** 10.1192/j.eurpsy.2025.1844

**Published:** 2025-08-26

**Authors:** S. Mashegoane, J. O. August, V. Baloyi, L. D. M. Lebeloane, M. Makhubela, K. P. Moalusi

**Affiliations:** 1 Psychology, University of Limpopo, Polokwane; 2 Psychology, Nelson Mandeal University, Qheberha; 3 Psychology, University of Venda, Thohoyandou; 4 Further Teacher Education; 5 Industrial and Organisational Psychology, University of South Africa, Tshwane, South Africa

## Abstract

**Introduction:**

COVID-19 wreaked havoc across the world killing millions along its path. All attempts were made to lower and eventually control the death toll from the pandemic. The “trace, test and treat” approach had its limits since the latter were not developed fast enough. Vaccines were seen as the best hope to protect individuals from the coronavirus and COVID-19. Thus, vaccination was encouraged and promoted widely. Aside from vaccines, interventions emphasised non-pharmaceutical self-protective behaviours to protect against coronavirus infection. Subsequently, there were various levels of compliance and observance of self-protection within nations. Yet studies have not attempted to explore the research implications of compliance patterns.

**Objectives:**

The present study’s aim was to (i) identify latent classes of individuals’ varying levels of compliance with COVID-19 self-protective behaviours; and (ii) explore the capacity of the latent classes to separate individuals according to their levels of Perceived Infectability, Germ Aversion and Fear of COVID-19.

**Methods:**

Data for the current study was extracted from a cross-district COVID-19 study conducted among *high school level learners (N = 1609; girls = 59%; rural areas = 43%) in South Africa.* Latent classes were derived based on the scores obtained by learners on a self-developed index of non-pharmaceutical self-protective behaviours. Three classes were identified, and they were compared against their obtained Perceived Infectability, Germ Aversion and Fear of COVID-19 scores.

**Results:**

Scores of all three knowledge groups did not differ on Perceived Infectability (p > .05), but the highest scorers, the “knowledgeable group”, scored higher than the “moderately knowledgeable group” and the “relatively low knowledge group” on Germ Aversion and Fear of COVID-19. The scores of the “moderately knowledgeable group” and the “relatively low knowledge group” did not differ on the Fear of COVID-19.

**Conclusions:**

The study supports an approach where learners are classified according to their knowledge of COVID-19 self-protective behaviours, and their motivation for self-protection established according to the classification.

**Disclosure of Interest:**

None Declared

